# ﻿A new species of *Cheironitis* van Lansberge, 1875 (Coleoptera, Scarabaeidae, Onitini) and the first record of *Cheironitis moeris* (Pallas, 1787) from China

**DOI:** 10.3897/zookeys.1265.174240

**Published:** 2025-12-30

**Authors:** YuHeng Wang, Olivier Montreuil, Paul Coppo

**Affiliations:** 1 Bingtuan No.2 Middle School, Xinjiang, China Bingtuan No.2 Middle School Xinjiang China; 2 MECADEV, UMR 7179 MNHN/CNRS, Muséum national d’Histoire naturelle, cp 50, F – 75231 Paris cedex 05, France MECADEV, Muséum national d’Histoire naturelle Paris France; 3 AP-HP-SU, Paris, France AP-HP-SU Paris France; 4 INSERM UMRS1138, Centre de Recherche des Cordeliers, Université Paris Cité, Sorbonne Université, Paris, France Centre de Recherche des Cordeliers, Université Paris Cité, Sorbonne Université Paris France

**Keywords:** Asia, China, dung beetle, Scarabaeinae, taxonomy, Xinjiang Uygur Autonomous Region

## Abstract

A new species *Cheironitis* van Lansberge, 1875 (*Cheironitis aiweiae***sp. nov.**), is described from Shiren Gou, Urumqi, in the XinJiang Uygur Autonomous Region of China. *Cheironitis moeris* (Pallas, 1787) is also reported from this region. These two original observations complement the composition of *Cheironitis* species in China and further expand our knowledge of their distribution.

## ﻿Introduction

The genus *Cheironitis* Lansberge, 1875, contains 24 species occurring in relatively arid areas of Palaearctic and Afrotropical regions (Janssens 1937; Ferreira 1960, 1976; Balthasar 1963a, 1963b; Martín Piera 1987; Baraud 1992; Kabakov 2006; Bezděk 2016; Coppo and Montreuil 2021). The *Cheironitis* fauna of China is poorly studied. Kabakov (2006) recorded only *C. eumenes* (Gebler, 1860) with certainty, while he suspected the presence of *C. haroldi* (Ballion, 1871) and *C. moeris* (Pallas, 1787) in this country.

The study of new material helps to clarify the species composition of the Chinese dung beetle fauna.

## ﻿Material and methods

Specimens were collected from Shiren Gou, Xinjiang Uygur Autonomous Region, China on June 23, 2022; June 28, 2022; June 12, 2023; June 23, 2023; and August 12, 2023, and are currently deposited at the Xinjiang Institute of Biogeography. These specimens were compared with the most closely related Palearctic species. Dry specimens were examined using a Lenovo Zhaoyang E5-ITL laptop, and a Canon R10 camera was used to capture images. The aedeagus of the new species was observed under a WST-2KCH microscope, and all resulting images were edited using Adobe Photoshop 2023.

To confirm the identification of our specimens as *Cheironitis moeris* (Pallas, 1787), they were compared with a specimen available at the **MNHN** (Muséum national d’Histoire naturelle, Paris, France) (locality: Sarepta [now Volgograd], Russia). *Cheironitis aiweiae* sp. nov. was compared with its closest species, *C. haroldi* (Ballion, 1871) (examined material from the MNHN, Paris, France).

## ﻿Taxonomy

**Coleoptera**

**Scarabaeidae**

### ﻿ Cheironitis moeris

Taxon classificationAnimaliaColeopteraScarabaeidae

(Pallas, 1787)

976A571F-DEE0-504B-991A-1ACD5BFC84E6

Fig. 1

#### Material examined.

**China** • Shiren Gou, YuHeng Wang leg., 43°49'41"N, 87°46'02"E, 892 m, 23 Jun. 2022, adult male, det. Olivier Montreuil; • Shiren Gou; YuHeng Wang leg.; 43°49'41"N, 87°46'02"E, 903 m, 28 Jun. 2022, adult male, det. Olivier Montreuil; • Shiren Gou, YuHeng Wang leg., 43°49'41"N, 87°46'02"E, 903 m, 28 Jun. 2022, adult female; det. Olivier Montreuil; • Manas County, FanHao Zen leg., 43°48'17"N, 87°38'09"E, 446 m, 12 Jun. 2023; adult male; det. Olivier Montreuil; • Shiren Gou, YuHeng Wang leg., 43°49'41"N, 87°46'02"E, 912 m, 12 Aug. 2023, adult male; det. Olivier Montreuil. These five specimens are deposited in YuHeng Wang’s personal collection.

**Figure 1.**
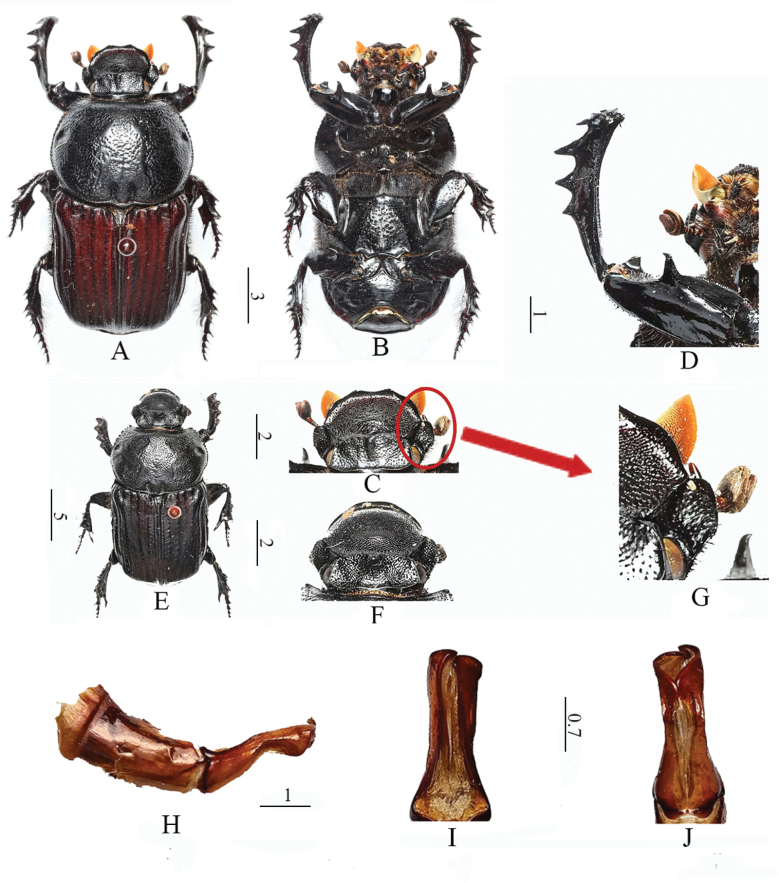
*Cheironitis moeris* (Pallas, 1787). **A.** Habitus male dorsal view; **B.** Ventral view of male; **C.** Head of male; **D.** Male protibia; **E.** Habitus female dorsal view; **F.** Head of female; **G.** Detail of male gena; **H.** Right lateral view of aedeagus; **I.** Ventral view of parameres; **J.** Dorsal view of parameres. Scale bars: 0.7 mm (**I, J**); 1.0 mm (**D, H**); 2.0 mm (**C, F**); 3.0 mm (**A, B**); 5.0 mm (**E**).

#### Remarks.

*Cheironitis moeris* (Pallas, 1787) is a species known from Central Asia with a distribution that extends westward to Turkmenistan and northward to Russia (Kabakov 2006). Here, we provide definitive evidence that *C. moeris* (Pallas, 1787) belongs to the China fauna, extending its eastward area of distribution, which overlaps with that of *C. osiridis* (Reiche, 1856) and *C. haroldi* (Ballion, 1871).

#### Distribution.

Russia, Kyrgyzstan, Kazakhstan, Armenia, Afghanistan, Uzbekistan, China.

### ﻿ Cheironitis aiweiae

Taxon classificationAnimaliaColeopteraScarabaeidae

YuHeng Wang, Paul Coppo & Olivier Montreuil sp. nov.

0A444A12-BBDD-5579-89E7-1F81881D3FD7

https://zoobank.org/42FE0151-092F-461B-A9F6-56DE3DE6EFDB

Fig. 2

#### Holotype.

**China** • Shiren Gou, YuHeng Wang leg., 43°49'34"N 87°46'06"E, 885 m, 23 Jun. 2022; adult male; collection ID: XJBI(E)0050002 (Museum of Xinjiang Institute of Ecology and Geography, Chinese Academy of Sciences (XJBI))**. *Paratype***: • Shiren Gou, YuHeng-Wang leg., 43°49'34"N 87°46'06"E, 889 m, 28 Jun. 2022; adult male; collection ID: YuHeng Wang’s personal collection, Xinjiang, China.

#### Description.

**Figure 2.**
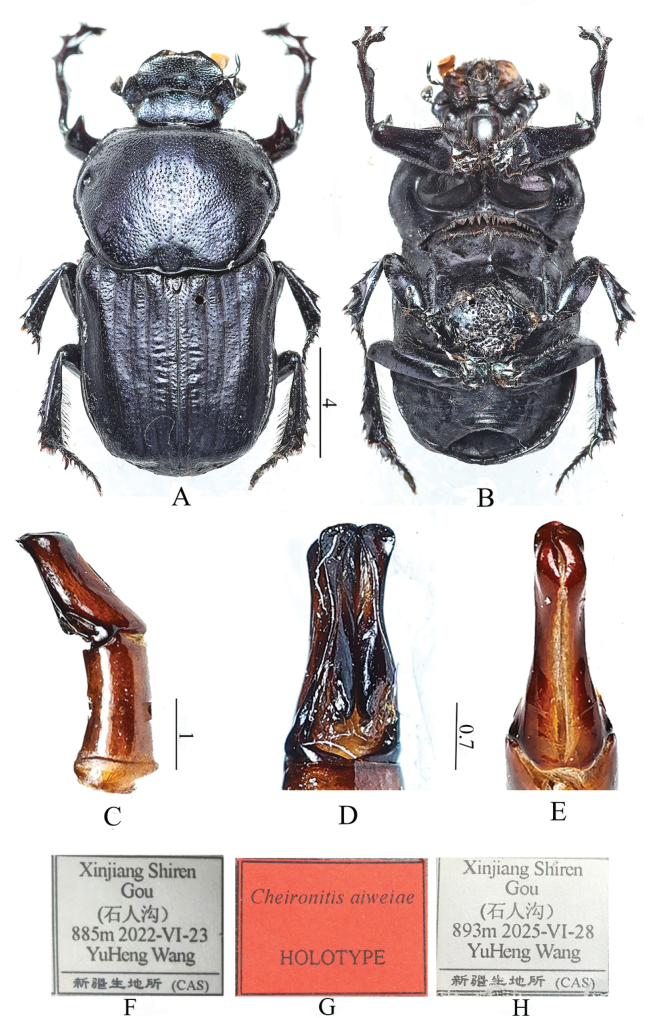
*Cheironitis aiweiae* sp. nov., holotype male, habitus. **A.** Dorsal view; **B.** Ventral view; **C.** Right lateral view of aedeagus; **D.** Ventral view of parameres; **E.** Dorsal view of parameres; **F, G.** Holotype label; **H.** Paratype label. Scale bars: 0.7 mm (**D, E**); 1.0 mm (**C**); 4.0 mm (**A, B**).

**Holotype** (Fig. 2A). **Male: *Overall aspect*.** Length 19.5 mm. Body dark blue with metallic luster, and black setae (Fig. 2A). ***Head*.** Wide with irregular punctations. Clypeus semicircular anteriorly, strongly incurved in the middle. Frontal carina slightly curved. Clypeal carina shorter than the space between the clypeal teeth. Circumocular ridge very apparent. ***Pronotum*.** Width longer than length. Deep concave points on both sides. Outer edge noticeably wider in the middle than at the two ends of the external margin. Pronotal margins coarsely serrated. Two adjacent deep foveae at the posterior end of pronotum. Two foveae on both sides of the midline at the median-posterior portion of the pronotum. ***Elytra*.** Slightly shorter than pronotal width. Lateral carina strong on first half and faint thereafter. Elytral suture prominent. Multiple irregular granules on the surface. Lateral carina weak on first half and vanishing thereafter. ***Legs*.** Protibia developed, with 4 tooth-like protrusions on the external margin, the proximal tooth very small. Protibia curved downward at 2/3 and inward thereafter. Two teeth on internal margin of protibia, at the same level as first and third teeth on external margin; multiple setae on internal margin of distal area. Profemora black, with dense setae on internal margin. Two spines at the junction of profemora and protibia, one dorsally-directed and one ventrally-directed. Dorsally-directed spine strongly developed (Fig. 2A) ***Ventral side*.** Mesosternum black, densely and irregularly punctuated. Propleuron smooth (Fig. 2B) ***Adeagus*.** Parameres and phallobase of almost equal length. Distal end of parameres uncinate. Bulge on the 1/4 of ventral surface (Fig. 2C–E).

**Paratype**, male. Body length is 19.7 mm, otherwise similar to the holotype.

**Female**: unknown.

#### Distribution.

Xinjiang Uygur Autonomous Region, China.

#### Etymology.

This species is named after the first author’s mother’s name.

#### Diagnosis.

The new species *Cheironitis aiweiae* sp. nov. belongs to the black species group of *Cheironitis* inhabiting Central Asia and surrounding areas: *C. haroldi* (Ballion, 1871), *C. osiridis* (Reiche, 1856), *C. klapperichi* (Balthasar, 1955), *C. furcifer* (Rossi, 1792), *C. moeris* (Pallas, 1787), *C. sterculius* (Ballion, 1871), and *C. candezi* (Lansberge, 1875). In addition to the special curve of the protibia, the new species can be clearly distinguished from these related species by the following obvious characteristics:

*Cheironitis furcifer* (Rossi, 1792): Protibia with only three teeth. Anterior margin of male profemora deeply emarginated, with a strong, complicated, forward-pointing lamella. Prosternal process strongly protrusive, forming a two-spined fork.

*Cheironitis moeris* (Pallas, 1787): Pronotum with raised, unpunctured, smooth areas. Anterior margin of male protibia with a strong forward-pointing tooth separated from apical edge.

*Cheironitis sterculius* (Ballion, 1871) and *C. candezi* (Lansberge, 1875): Both larger than the new species and have strong teeth in the middle of the underside of the protibia.

*Cheironitis osiridis* (Reiche, 1856): Anterior margin of male protibia with one long downward-pointing tooth near the base.

*Cheironitis klapperichi* (Balthasar, 1955): Anterior margin of male protibia with one strong forward-pointing fork-shaped process near the base.

**Figure 3.**
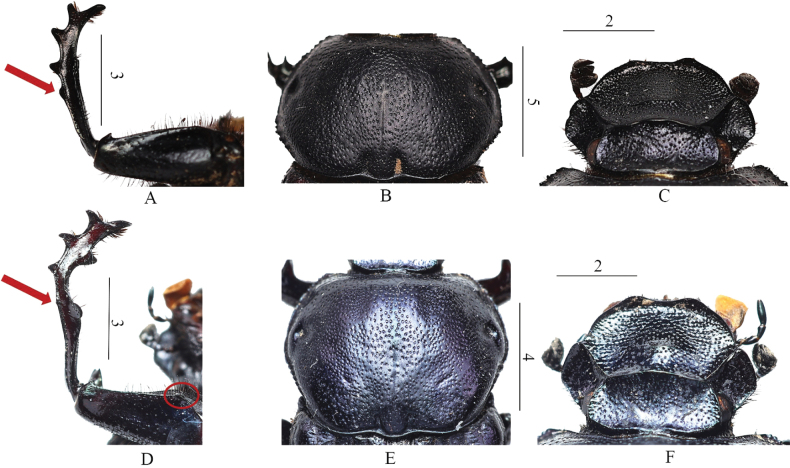
Details of protibia, pronotum and head of *Cheironitis haroldi* (Ballion, 1871) (**A, B, C**) and *Cheironitis aiweiae* sp. nov. (**D, E, F**). Red arrows show the difference in the apical tooth at the anterior margin of the male protibia between both species. The red circle shows the tooth at the base of the anterior margin of the male profemora in *Cheironitis aiweiae* sp. nov. Scale bars: 2.0 mm (**C, F**); 3.0 mm (**A, D**); 4.0 mm (**E**); 5.0 mm (**B**).

By the elytra distinctly and completely covered by small granules and the lack of an apical tooth at the anterior margin of the male protibia (Fig. 3; arrows), the new species seems closer to *C. haroldi* (Ballion, 1871). The main differences between the two species are summarized in Table 1. As *Cheironitis* species are known to have significant allometric variability in the male’s leg shapes, the differences presented here are mostly relevant to major males.

**Table 1.** Main differences between *Cheironitis aiweiae* sp. nov. and its closest species *C. haroldi*.

Morphological features	*Cheironitis haroldi* (Ballion, 1871)	*Cheironitis aiweiae* sp. nov.
Prosternal process	One inconspicuous tooth	No tooth
Apical dorsal margin of male profemora	With a short, simple tooth (Fig. 3A)	One long tooth dorsally-directed divided at apex, and one short tooth ventrally-directed (Fig. 3D)
Male protibiae	Four teeth on the external margin, the proximal smaller. Inner margin shows a gradually curved profile (Fig. 3A)	Four teeth on the external margin, the proximal very small. Protibia curved downward at 2/3 and abruptly strongly inward thereafter (Fig. 3D)
Pronotum	Four foveae on surface (Fig. 3B)	Six foveae on surface. Two foveae on both sides of the midline at the median-posterior portion of the pronotum (Fig. 3E)
Male clypeal carina	Long, as long as the space between the clypeal teeth (Fig. 3C)	Shorter; shorter than the space between the clypeal teeth (Fig. 3F)
Base of anterior margin of male profemora	With inconspicuous tooth	With one short but obvious tooth (Fig. 3D)
Distribution	Middle East to Central Asia, China	Shiren Gou, Xin Jiang, China

##### ﻿Natural history

**Figure 4.**
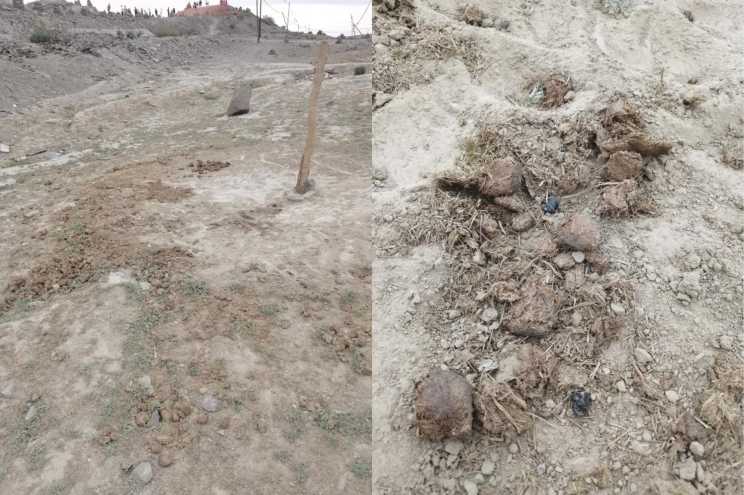
Biotope of *C. aiweiae* sp. nov.

A few specimens of *Cheironitis aiweiae* sp. nov. were collected from horse dung in June in arid areas (Fig. 4), consistent with the relatively dry habitat of other species in the genus.

This new species was found in association with only *C. moeris* (Pallas, 1787). No other Scarabaeinae species were found in association with these two species, probably because of the particularly dry condition of the habitat during the period of the year when the active beetles were collected.

## Supplementary Material

XML Treatment for Cheironitis moeris

XML Treatment for Cheironitis aiweiae

## ﻿Additional information

### Use of AI

No use of AI was reported.

### Author ORCIDs

Olivier Montreuil https://orcid.org/0000-0003-2682-7594

Paul Coppo https://orcid.org/0000-0002-4618-2095

### Data availability

All of the data that support the findings of this study are available in the main text.
